# Effects of variety, year of cultivation and sulphur supply on the accumulation of free asparagine in the grain of commercial wheat varieties

**DOI:** 10.1016/j.foodchem.2017.06.113

**Published:** 2018-01-15

**Authors:** Tanya Y. Curtis, Stephen J. Powers, Ruiyun Wang, Nigel G. Halford

**Affiliations:** a Plant Science Department, Rothamsted Research, Harpenden, Hertfordshire AL5 2JQ, United Kingdom; b Computational and Analytical Sciences Department, Rothamsted Research, Harpenden, Hertfordshire AL5 2JQ, United Kingdom; c Agronomy College, Shanxi Agricultural University, and Key Laboratory of Crop Gene Resources and Germplasm Enhancement on Loess Plateau, Ministry of Agriculture, Shanxi Key Laboratory of Genetic Resources and Genetic Improvement of Minor Crops, Institute of Crop Germplasms Resources of Shanxi Academy of Agricultural Sciences, Taiyuan City, Shanxi Province 030031, People’s Republic of China

**Keywords:** Acrylamide (PubChem CID: 6579), l-Asparagine (PubChem CID: 6267), Acrylamide, Asparagine, Food safety, Free amino acids, Processing contaminants, Wheat

## Abstract

Free asparagine concentration, which is the determining factor for acrylamide-forming potential in cereals, was measured in grain from wheat grown in field trials in the United Kingdom in 2011–2012 and 2012–2013. There were 25 varieties in 2012 and 59 in 2013, with eleven present in both trials. The trials were split-plot, with half of each plot supplied with sulphur and the other half not. The varietal means (mmol per kg) for free asparagine in the sulphur-fed wheat ranged from 1.521 to 2.687 in 2011–2012 and 0.708 to 11.29 in 2012–2013. Eight varieties were identified as having consistently low free asparagine concentration. There was a differential response of varieties to sulphur, and much higher levels of free asparagine in 2012–2013 versus 2011–2012. Given the short commercial lifespan of some wheat varieties, it is concluded that information on free asparagine concentration should be made available when a variety is launched.

## Highlights

• Widely differing free asparagine concentration in wheat from two harvest years.
• Wide range of acrylamide-forming potential (AFP) in 73 commercial wheat varieties.
• Eight varieties identified with consistently low AFP, seven of them soft types, but benefit of using a low AFP variety is lost if sulphur supply is inadequate.
• Selecting varieties for low AFP solely on the basis of them being soft would be simplistic.
• Information on free asparagine concentration should be available at variety launch.

## 1 Introduction

Food processing and cooking bring about substantial changes in food composition, including the production of substances that are not present in the raw food. These changes may be necessary to make the food edible and/or palatable, and many of the substances that are produced are responsible for the colours, flavours, and aromas that define food types and distinguish brands. However, some of the substances that are produced fall into the category of processing contaminant, defined as a substance that is produced in a food when it is cooked or processed, is not present or is present at much lower concentrations in the raw, unprocessed food, and is undesirable either because it has an adverse effect on product quality or because it is potentially harmful (Curtis, Postles, & Halford, 2014).

A processing contaminant that is proving to be an increasingly difficult problem for the food industry is acrylamide, which forms within the Maillard reaction during the frying, baking, roasting or high-temperature processing of cereals, potatoes, coffee and other plant-derived raw materials, with all major cereal products, including bread, crispbread, breakfast cereals, cakes and biscuits, being affected (European Food Safety Authority, 2011). Acrylamide is classed as a probable (Group 2a) human carcinogen by the International Agency for Research on Cancer (1994), based on its action in rodents, and also has reproductive and neurotoxicological effects at high doses (Friedman, 2003).

The European Food Safety Authority (EFSA) Expert Panel on Contaminants in the Food Chain (CONTAM) has stated that it considers the margin of exposure for acrylamide (the ratio of the level at which a small but measurable effect is observed to the estimated exposure dose) to be low enough to cause concern that dietary acrylamide could have neoplastic (tumor-inducing) effects (European Food Safety Authority Panel on Contaminants in the Food Chain (CONTAM), 2015). As a result, the European Commission is reviewing its risk management measures for dietary acrylamide intake, which since 2011 have been based on non-mandatory ‘Indicative Values’ for the presence of acrylamide in foods (European Commission, 2013). In the USA, the Food and Drug Administration (FDA) has so far stopped short of issuing advice or restrictions on levels of acrylamide in food, but has issued an ‘action plan’ for the food industry (Food and Drug Administration, 2016).

Acrylamide forms predominantly *via* a Strecker-type degradation of free (i.e. soluble, non-protein) asparagine by highly reactive carbonyl compounds produced within the Maillard reaction (Mottram et al., 2002, Stadler et al., 2002, Zyzak et al., 2003), although other routes for its formation have been proposed (Claus et al., 2006, Granvogl and Schieberle, 2006). The production of carbonyl compound intermediates within the Maillard reaction also involves reducing sugars, such as glucose, fructose and maltose, and other free amino acids, which means that the concentrations of these metabolites as well as free asparagine may affect acrylamide formation, depending on their relative concentrations (Muttucumaru et al., 2017). However, in wheat (*Triticum aestivum*) and rye (*Secale cereale*), and probably other cereals, free asparagine concentration is the major determinant of acrylamide-forming potential (Curtis and Halford, 2016, Curtis et al., 2016, Curtis et al., 2009, Curtis et al., 2010, Granvogl et al., 2007, Muttucumaru et al., 2006, Postles et al., 2013).

The challenge for the food industry is to reduce acrylamide levels while retaining the colours, flavours and aromas that define products and brands and are demanded by consumers. The methods that the industry has developed to reduce acrylamide formation have been shared in the ‘Acrylamide Toolbox’, published by FoodDrinkEurope (2013). For cereal-based products, the Toolbox recommends the use of grain with low free asparagine content, and switching from a high free asparagine to a low free asparagine variety would be easier and cheaper than doing anything else, while any change to processing would be more effective from a low asparagine starting point. However, this is not as simple as it sounds, because free asparagine concentration in many plant tissues, including cereal grain, is affected by environmental factors (E) that are unpredictable and beyond the control of producers (Halford et al., 2015, Lea et al., 2007). Nevertheless, crop management measures have been shown to be effective, and in the case of wheat this means ensuring that the crop is supplied with sufficient sulphur during cultivation and is protected from disease (Curtis and Halford, 2016, Curtis et al., 2009, Curtis et al., 2014, Curtis et al., 2016, Granvogl et al., 2007, Martinek et al., 2009, Muttucumaru et al., 2006). Genetic factors (G) also play a part, on their own and interacting with the environment (G × E) (Corol et al., 2016, Curtis et al., 2009, Curtis et al., 2010, Curtis et al., 2016, Curtis and Halford, 2016, Granvogl et al., 2007, Muttucumaru et al., 2006, Postles et al., 2013), and key questions for wheat breeders as they begin to address the acrylamide issue include: 1) how wide is the range in free asparagine concentration in different wheat genotypes? and 2) Is it possible to identify varieties that are consistently low in free asparagine concentration in the grain in a range of environments? In the present study we address these questions by comparing elite wheat varieties from the UK, grown in field trials in 2011–2012 and 2012–2013, with and without sufficient sulphur.

## 2 Materials and methods

### 2.1 Field trials

Field trails of winter wheat were carried out at the Rothamsted Farm site at Woburn, Bedfordshire, United Kingdom (UK), in 2011–2012 and 2012–2013. There were 73 varieties and genotypes altogether over the two trials, 25 in 2011–2012 and 59 in 2012–2013, with 11 varieties being present in both trials. The trials comprised a split-plot in three randomized blocks (replicates) design with sulphur applied to one half of each plot and not the other. In the 2012–2013 trial, genotype SR3, which had previously been shown to have a relatively low free asparagine concentration in the grain, was replicated twice in each block. The 2011–2012 trial was fertilized with Nitram® (ammonium nitrate: CF Fertilisers, Chester, UK) on the 28th of March 2012 at a rate of 116 kg/ha (40 kg/ha nitrogen), and on the 10th of May 2012 at a rate of 262 kg/ha (90 kg/ha nitrogen). Sulphur was applied by hand on the 23rd of April 2012 as agricultural gypsum (calcium sulphate dihydrate) (Saint-Gobain, British Gypsum, Loughborough, UK) at a rate of 40 kg sulphur per hectare (100 kg per hectare SO_3_ equivalent). Planting of the 2012–2113 trial was delayed due to heavy rain, and Nitram® was not applied until 25th April 2013, at a rate of 174 kg/ha (60 kg/ha nitrogen), and then on the 9th of May 2013 at a rate of 232 kg/ha (80 kg/ha nitrogen). Sulphur was again applied by hand on the 26th of April 2013 as agricultural gypsum at a rate of 40 kg sulphur per hectare. Grain was harvested in August 2012 and 2013, and samples milled to fine, wholemeal flour for analysis.

### 2.2 Free amino acid concentrations

Flour (0.5 g ± 0.005 g) was added to 10 mL of 0.01 N HCl and stirred for 15 min. The suspension was left to settle for 15 min at room temperature and an aliquot (1.5 mL) was centrifuged at 7200*g* for 15 min to produce a clear extract. Amino acids were derivatised using the EZ: Faast free amino acid kit (Phenomenex, Torrance, CA) using the protocol provided in the manufacturer’s manual with the following modifications: a second wash step was included with Reagent 2, and the sample was vortexed for 10 s after addition of Reagent 4, then rested for 2 min before vortexing again for 16 s before derivatisation. Gas chromatography-mass spectrometry (GC–MS) analysis of the derivatised samples was carried out using an Agilent 6890 GC-5975-MS system (Agilent, Santa Clara, CA) in electron impact mode, as described previously (Elmore, Koutsidis, Dodson, Mottram, & Wedzicha, 2005). An aliquot of the derivatised amino acid solution (1 μL) was injected at 250 °C in split mode (20:1) onto a Zebron ZB-AAA capillary column (10 m × 0.25 mm; 0.25 μm film thickness). The oven temperature was held at 110 °C for 1 min and then increased at 30 °C min^−1^ to 310 °C. The transfer line and ion source were maintained at 320 °C and 230 °C respectively; carrier gas flow rate was kept constant throughout the run at 1.1 mL min^−1^. Amino acid standards were provided with the EZ: Faast kit and were >99% pure (Phenomenex). Separate calibration curves were calculated for each amino acid. The standards were also used before, during and after the analysis of each batch of 200 samples to check that the machine was running correctly. Two technical replicates per biological replicate sample were assayed for the 2011–2012 trial, and three per biological replicate sample for the 2012–2013 trial. Analyses of the data to extract quantities (mmol/kg) of each amino acid using the calibration curves were performed using the Agilent 5975 system data analysis software.

### 2.3 Statistical analyses

Due to the imbalance caused by the different sizes of the field trials, and the fact that some but not all varieties were included in both trials, the method of residual maximum likelihood (REML) was used to fit a linear mixed model to the data from both trials. The design terms of blocks within trials, main-plots within blocks, and split-plots within main plots were taken as random terms (variance components) and the main effects and interactions between the factors of year, variety, type (group) and sulphur treatment were taken as fixed terms. These latter terms were tested using approximate F-tests. Appropriate predicted means and standard error of the difference (SED) values were output along with the least significant difference (LSD) values for comparison of means, noting that here it makes sense to predict means only for the 11 varieties common to both trials, with the results from the individual trials being more relevant for the other varieties. Hence, analysis of variance (ANOVA) was applied to data from the individual trials, but with interpretation of the results (using F-tests and SED/LSD values) being the same as for application of the REML method. For both methods, the log_e_ transformation of the amino acid data was used to account for some heterogeneity of variance across the variety by treatment combinations, and thus to ensure conformation to the assumptions of the analysis. The GenStat (2015, 18th edition, VSN International Ltd, Hemel Hempstead, UK) statistics package was used for the analyses.

## 3 Results and discussion

### 3.1 Free asparagine levels in wheat genotypes grown in field trials in 2011–2012 and 2012–2013

Field trials of a range of winter wheat genotypes were performed over two years from 2011 to 2013. There were 73 varieties altogether over the two trials, with 11 varieties being present in both. The majority of the varieties were or had been on the UK’s Agriculture and Horticulture Development Board (AHDB; previously known as the Home Grown Cereals Authority) Recommended List of varieties for commercial cultivation (https://cereals.ahdb.org.uk/varieties/ahdb-recommended-lists.aspx). The varieties and genotypes per trial (25 in 2011–2012 and 59 in 2012–2013) are listed in Table 1, grouped according to their classification by the National Association of British and Irish Millers (NABIM) (http://www.nabim.org.uk/wheat/wheat-varieties). These groups are: Group 1, varieties with consistent milling and baking performance; Group 2, varieties with bread-making potential but not suited to all grists; Group 3, soft varieties used for biscuits, breakfast cereals, cakes and similar products; Group 4, sub-grouped into hard and soft types; used mainly for animal feed and bioethanol, but incorporated into some grists for food use.

**Table 1:** Varieties and genotypes used in the 2011–2012 and 2012–2013 field trials, grouped according to their NABIM classification (http://www.nabim.org.uk/wheat/wheat-varieties).

Group	2011–2012		2012–2013	
Bread G1	Avalon	Shamrock	Avalon	Skyfall
	Gallant	Solstice	Crusoe	Solstice
	Hereward	Spark	Gallant	Spark
	Malacca			

Bread G2	Cadenza		Bonham	Einstein
	Charger		Cadenza	Evoke
	Rialto		Cashel	Podium
	Shango		Chilton	Rialto
			Cordiale	Sterling
			Cubanita	

Biscuit G3	Claire	Scout	Cocoon	Invicta
	Delphi	Torch	Croft	Monterey
	Diego	Tuxedo	Delphi	Torch
	Invicta	Warrior	Diego	Weaver
	Robigus		Icon	Zulu

Soft G4			Alchemy	Myriad
			Cougar	Panacea
			Dali	Revelation
			Denman	Rowan
			Horatio	Twister
			Lancaster	Viscount
			Leeds	

Hard G4	Badger		Dickens	Icebreaker
	Buster		Duxford	Kielder
	Oakley		Evolution	Relay
	Santiago		Gator	Santiago
	Savannah		Goldengun	Solace

Other			SR3	AxC104
			AxC100	AxC201
			AxC101	AxC202
			AxC102	AxC205
			AxC103	

The variety list for 2011–2012 comprised seven Group 1 varieties, four Group 2, nine Group 3 and five Group 4 (hard), while those grown in the 2012–2013 field trial comprised six Group 1 varieties, eleven Group 2, ten Group 3, thirteen Group 4 (soft) and ten Group 4 (hard) (Table 1). Nine other genotypes were also included in 2012–2013: SR3, a doubled haploid line from a Spark × Rialto mapping population, which had previously been shown to have a relatively low free asparagine concentration in the grain (Curtis et al., 2009), and eight doubled haploids from an Avalon × Cadenza mapping population (Ma et al., 2015) (denoted A × C100 – A × C104, A × C201, A × C202 and A × C205). Both trials used a split-plot design, with nitrogen being supplied to main plots of varieties and sulphur being supplied to one half of each main plot but not the other.

Data for free amino acid concentrations on the log_e_ and raw scale in wholemeal flour produced from grain harvested from the trials are given in Supplementary File S1 (apart from arginine, which could not be measured by the method used), the log_e_ transformation being used for statistical analysis of the data. ANOVA was applied to the data to investigate the effects of treatment (sulphur application), type (NABIM group), variety within type and the interactions between them. The results are given in Table 2.

**Table 2:** The p-values from the analysis of variance (ANOVA) to consider the significance of treatment, wheat type, variety nested in type, and the interactions between these factors for the 2011–2012 and 2012–2013 field trials. The values in bold are significant (p < 0.05, F-test), whereas those in bold and underlined indicate the ANOVA terms to be interpreted, these superseding the ones only in bold.

Amino Acid	Treatment	Type	Type.Variety	Type.Treatment	Type.Variety.Treatment
2011–2012					
Ala	0.901	0.072	**<0.001**	0.258	0.362
Asn	**0.040**	**0.042**	**<0.001**	0.274	**0.062**
Asp	0.527	**<0.001**	**<0.001**	0.175	0.379
Cys	0.353	0.276	0.738	0.094	0.736
Gly	0.559	**<0.001**	**<0.001**	0.973	0.155
Gln	0.812	0.740	**<0.001**	0.375	0.770
Glu	0.825	0.662	**<0.001**	0.101	0.071
His	0.480	**0.040**	0.131	0.625	0.498
Leu	0.349	0.562	**<0.001**	0.292	0.427
Lys	0.962	0.351	**<0.001**	0.378	0.398
Ile	0.215	0.128	0.083	0.581	0.783
Met	0.420	0.918	0.209	0.789	0.147
Phe	0.653	**0.003**	**<0.001**	0.417	0.430
Pro	0.260	**<0.001**	**<0.001**	0.582	0.231
Ser	0.613	0.301	**<0.001**	0.242	0.143
Thr	0.744	0.124	**<0.001**	0.304	0.555
Trp	**0.032**	**0.032**	**<0.001**	0.211	0.122
Tyr	0.489	**<0.001**	**<0.001**	0.189	0.293
Val	0.353	0.213	**<0.001**	0.617	0.349
Unk	0.962	0.351	**<0.001**	0.378	0.398

2012–2013					
Ala	**0.040**	0.446	**0.004**	0.448	0.228
Asn	**0.007**	0.301	**0.002**	**<0.001**	**0.002**
Asp	**0.022**	0.205	**0.041**	0.468	0.342
Cys	0.431	0.931	0.070	**0.005**	0.396
Gln	**0.018**	0.828	0.101	**0.048**	0.574
Glu	0.068	0.096	**<0.001**	0.239	0.391
Gly	0.061	0.708	**<0.001**	**0.025**	0.167
His	0.361	0.347	**0.024**	**0.020**	0.120
Ile	0.166	**0.032**	**<0.001**	0.247	0.073
Leu	0.409	0.101	**<0.001**	0.466	0.101
Lys	**0.032**	0.702	**0.008**	**0.003**	0.682
Met	0.063	0.546	**0.002**	0.660	0.638
Phe	0.162	**<0.001**	**<0.001**	0.281	0.367
Pro	**0.039**	**<0.001**	**<0.001**	**0.008**	**0.043**
Ser	0.083	0.472	0.057	0.271	0.387
Thr	0.085	**0.048**	**<0.001**	**0.031**	**0.048**
Trp	**0.010**	**<0.001**	**<0.001**	0.797	0.441
Tyr	**0.029**	0.283	**<0.001**	**<0.001**	**0.029**
Val	0.255	0.407	**0.015**	0.179	0.248

For free asparagine in the 2011–2012 trial, the full interaction between variety nested in type and treatment was of marginal significance (p = 0.062, F-test) and therefore important for investigation, but with there being considerable variation due to variety within type (p < 0.001, F-test, for the variety within type ANOVA term) and a main effect of treatment (p = 0.040, F-test). For other amino acids where there was significance, it was for the variety nested in type effect, with the exception of tryptophan, for which there was also a main effect of treatment, with lower levels of this amino acid being found in conditions of sulphur deprivation, and histidine, for which there was only a main effect of type. The means tables of interest can be found in Supplementary File S1.

Analysis of the 2012–2013 data showed an interaction between variety nested within type and treatment for free asparagine, and also for threonine, proline and tyrosine (p < 0.05, F-tests). There was a variety nested within type and a type by treatment effect for glycine, lysine and histidine, and a type by treatment interaction, only, for glutamine and cysteine. There was a variety nested in type effect, only, for valine, leucine, isoleucine, methionine, glutamine and phenylalanine, and with some evidence of this effect also for serine (p = 0.057, F-test). There was a main effect of treatment and an independent main effect of variety nested in type for alanine, aspartic acid and tryptophan.

### 3.2 Varietal differences

**Fig. 1:**
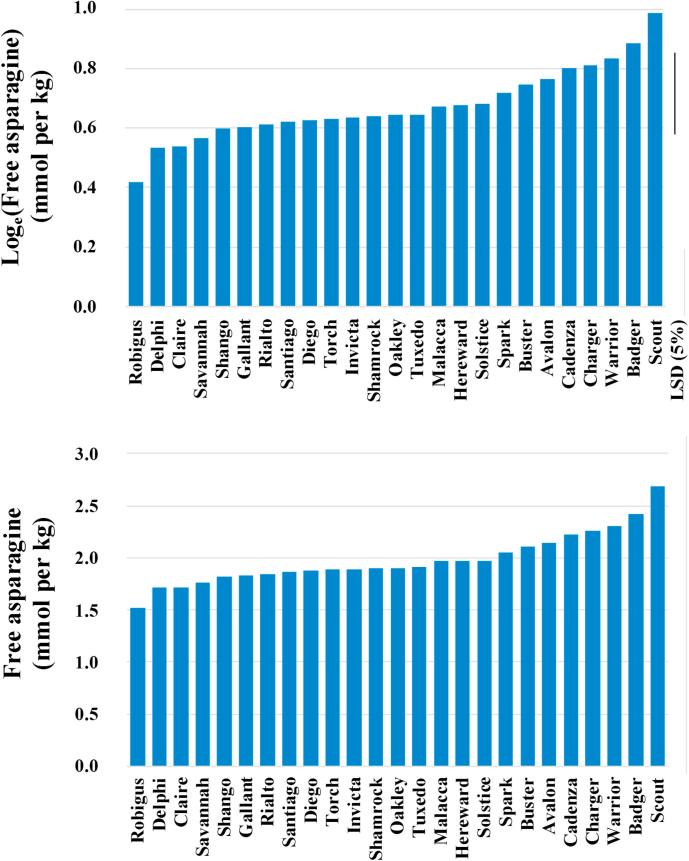
Graphs showing the mean free asparagine concentration on the log_e_ scale (top) and back-transformed raw scale (bottom) in the grain of 25 varieties of wheat grown in a field trial in 2011–2012, supplied with nitrogen at a rate of 130 kg per hectare (in two applications) and sulphur at 40 kg per hectare. The least significant difference (LSD) at the 5% level of significance on 95 degrees of freedom is shown in the top panel (*n* = 3).

The varietal means for asparagine in the sulphur-fed wheat from the 2011–2012 trial are plotted on the log_e_ and raw scale in Fig. 1, with the LSD at the 5% level of significance being shown alongside the log_e_ data to facilitate varietal comparisons. The range of free asparagine concentration was relatively narrow, from 1.521 mmol per kg in Robigus to 2.687 mmol per kg in Scout, a difference of 77% with respect to the lower figure. Robigus has been shown in a previous study to have a variable free asparagine concentration in the grain when grown at different sites in 2005–2006 and 2006–2007 (Curtis et al., 2009). That study also showed Claire to be consistently low in free asparagine across the different sites and years, and Claire was the third lowest variety in the 2011–12 dataset here. The second lowest variety for free asparagine concentration was Delphi, and this variety was also relatively low for free asparagine in a field trial conducted by Saaten Union at Cowlinge, Suffolk, UK in the same year (Curtis et al., 2016). It has also been reported by food industry sources to be consistently low for free asparagine concentration (Gavin Sharman, Weetabix Limited, Kettering, UK, personal communication).

**Fig. 2:**
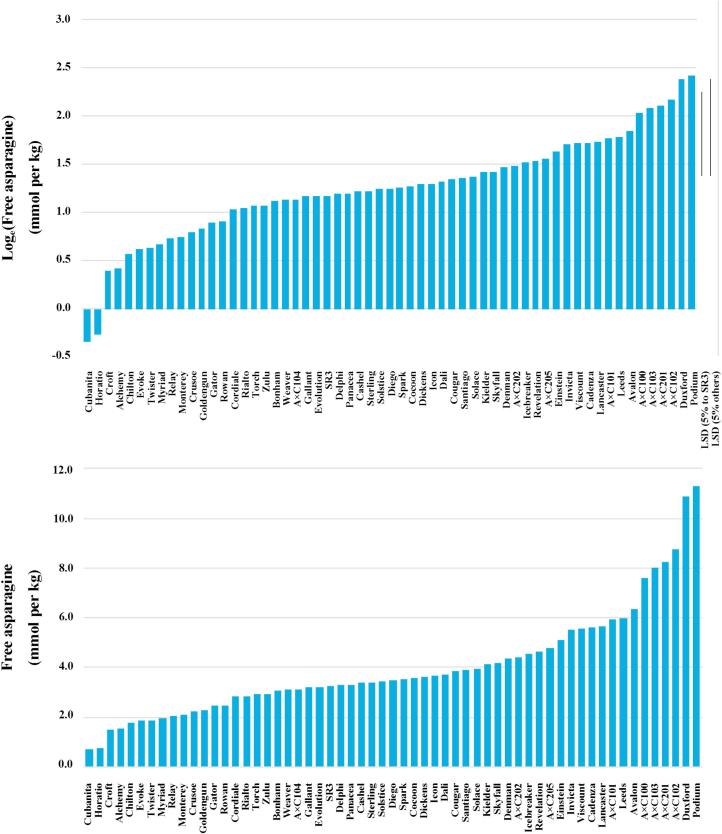
Graphs showing the mean free asparagine concentration on the log_e_ scale (top) and back-transformed raw scale (bottom) in the grain of 59 varieties/genotypes of wheat grown in a field trial in 2012–2013, supplied with nitrogen at a rate of 140 kg per hectare (in two applications) and sulphur at 40 kg per hectare. Least significant differences (LSD) at the 5% level of significance on 172 degrees of freedom for different comparisons, as indicated, are shown in the top panel (n = 6 for SR3, otherwise *n* = 3, hence the different LSDs).

The range of free asparagine concentrations in the sulphur-fed wheat from the 2012–2013 trial was much wider, from 0.708 mmol per kg for Cubanita to 11.29 mmol per kg for Podium (Fig. 2), a difference of almost 1500% with respect to the lower figure. Delphi was 3.296 mmol per kg compared with 1.709 mmol per kg in 2011–2012, but was still in the lower half of the variety rankings. No other data were available on Cubanita to enable its consistency to be assessed, but several varieties did emerge as being consistently low in this dataset, the dataset referred to above (Curtis et al., 2016) and in industry reports (Gavin Sharman, Weetabix Limited, Kettering, UK, personal communication): these were Horatio, Croft, Myriad, Monterey, Cordiale and Cocoon, the highest of which was Cocoon with 3.563 mmol per kg free asparagine, and the lowest Horatio with 0.765 mmol per kg. This suggests that, despite the fact that environmental factors (E) have a strong effect on free asparagine accumulation in wheat, on their own and in combination with genetic factors (G × E) (Curtis et al., 2009), it may be possible to identify some varieties that have low free asparagine concentration in the grain across a range of environments. This would be extremely useful for the food industry in selecting raw material that was reliably low in free asparagine concentration for its production lines. The free asparagine concentration in the Avalon × Cadenza doubled haploids ranged from 3.107 mmol per kg to 8.74 mmol per kg, while that of the Spark × Rialto doubled haploid SR3 was 3.24 mmol per kg.

### 3.3 Effects of wheat type and sulphur

**Fig. 3:**
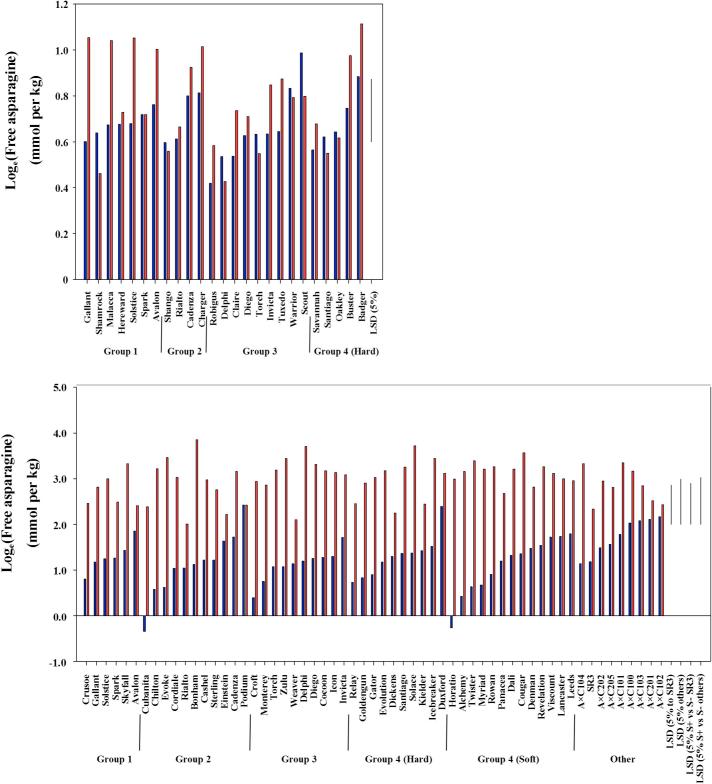
Graphs showing the mean free asparagine concentration on the log_e_ scale in the grain of 25 varieties of wheat grown in a field trial in 2011–2012 (top) and 59 varieties/genotypes grown in a field trial in 2012–2013 (bottom). Data are shown from split-plots in which wheat in half the plot was supplied with nitrogen and sulphur (S+; blue columns), while the wheat in the other half was supplied with nitrogen but not sulphur (S-; red columns). Least significant differences (LSD) at the 5% level of significance for different comparisons, as indicated, are shown (*n* = 3 for 2011–2012 on 96 degrees of freedom; *n* = 6 for SR3, otherwise *n* = 3 for 2012–2013 on 172 degrees of freedom, unless comparing treatments for SR3: 66 degrees of freedom, or for the other varieties/genotypes: 125 degrees of freedom). The varieties are shown separated into Groups 1 (breadmaking), 2 (breadmaking potential), 3 (biscuit) and 4, hard and soft (mainly animal feed and bioethanol), in ascending order for free asparagine concentration in the sulphur supplied condition within each group. (For interpretation of the references to colour in this figure legend, the reader is referred to the web version of this article.)

Of the eight varieties that emerged as being consistently low in free asparagine concentration, Delphi, Claire, Cocoon, Croft and Monterey are all soft Group 3 biscuit types, while Horatio and Myriad are soft Group 4 types. The eighth variety was Cordiale, which is Group 2, as is Cubanita, the variety with the lowest free asparagine concentration in the grain in the 2012–2013 field trial but for which other data were not available. In the 2011–2012 data there was a significant main effect of type (p = 0.042, F-test), with Group 3 biscuit wheats generally showing lower free asparagine concentration than the other types, although the full variety nested in type by treatment interaction (p = 0.062, F-test) is more appropriate for specific interpretation of varietal responses. However, in the 2012–2013 dataset, in which all types were represented, there was no main effect of type for free asparagine concentration (p = 0.301, F-test) (for types on average over the wheat lines within them and over the two treatments). The type by treatment (sulphur supply) interaction was significant (p < 0.001, F-test), suggesting that different types responded differently to the sulphur treatment, with Group 4 (soft) wheats most affected by sulphur deprivation. However, this was superseded by the full interaction of variety nested in type by treatment (p = 0.002, F-test), indicating that individual varieties within type responded differently to the treatment. The means tables for free asparagine and proline are given in Supplementary File S1, and the means for free asparagine for each trial, sorted from lowest to highest for the sulphur-supplied condition within each type, are plotted in Fig. 3.

A striking aspect of the data that is evident from the plots in Fig. 3 is the differential response to sulphur across the varieties, with the varieties with low free asparagine in the sulphur-supplied condition among those most affected by sulphur deprivation. This means that the varietal ranking for free asparagine concentration breaks down under conditions of sulphur deficiency.

### 3.4 Effects of harvest year

**Fig. 4:**
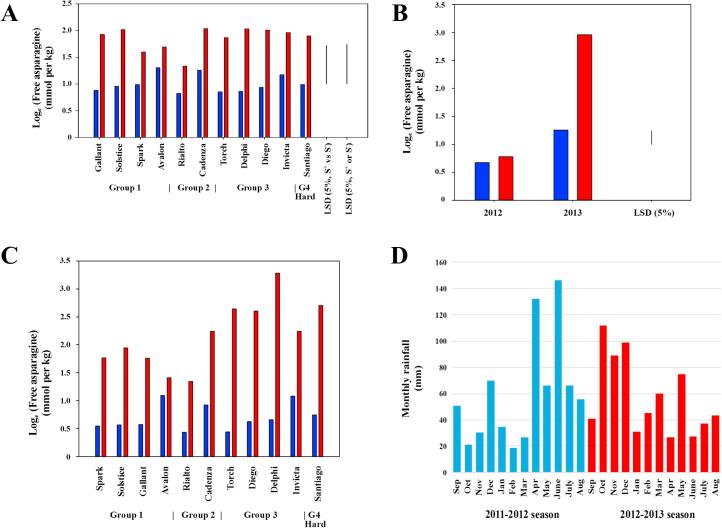
A and B. Graphs illustrating the results for interpretation from the linear mixed model (using REML) for free asparagine concentration in the grain of 11 varieties of wheat grown in field trials in both 2011–2012 and 2012–2013, with sulphur either supplied (blue columns) or withheld (red columns) for 2012 and 2013. A shows the mean (*n* = 6) free asparagine concentration on the log_e_ scale in the grain of the 11 varieties separately to illustrate the variety nested in type (Group) by treatment (sulphur supply) interaction (p = 0.015, F-test). Least significant differences (LSD) at the 5% level of significance on 266 degrees of freedom for different comparisons, as indicated, are shown. B shows the mean (*n* = 150 for 2011–2012 and *n* = 540 for 2012–2013) free asparagine concentration on the log_e_ scale for all the varieties together to illustrate the year by treatment interaction (p < 0.001, F-test). The least significant difference at the 5% level of significance on 266 degrees of freedom for comparison of means is shown. C. Graph showing differences (2013 minus 2012) in free asparagine concentration on the log_e_ scale to look at this aspect separately. Note that there was no full variety nested in type by year by treatment interaction, so there are no LSD values for this graph. D. Monthly rainfall at the field trial site, plotted from weather data provided by Dr. Sarah Perryman from the e-RA database, which is maintained as part of the Rothamsted Long-term Experiments National Capability (LTE-NCG). (For interpretation of the references to colour in this figure legend, the reader is referred to the web version of this article.)

Eleven varieties were grown in both trials, and the results from analyses by REML of the data for these varieties from the combined trials showed a significant variety nested in type (group) by treatment (sulphur supply) interaction (p = 0.015, F-test) (Fig.4A), and a significant year by treatment interaction (p < 0.001, F-test), illustrated in Fig.4B by showing the mean concentrations of all the varieties together. Not only were the free asparagine concentrations in the grain much lower in 2011–2012 than in 2012–2013, but they were much less responsive to sulphur deprivation, with no significant difference between the sulphur-supplied and -deprived grain in 2011–2012 (Fig.4B). The differences in free asparagine concentration (2012–2013 minus 2011–2012) are shown in Fig.4C: all eleven varieties were more stable from year to year when sulphur was supplied, but Delphi had one of the smallest year-on-year differences when sulphur was supplied but the largest when sulphur was deficient.

Relatively low free asparagine concentration and an unusual lack of response to sulphur supply in wheat grown in the UK in 2011–2012 has been reported previously (Curtis, Halford et al., 2014). One possible explanation for the difference between the two years is that 2012 had an exceptionally wet spring and summer. The monthly rainfall at the Woburn farm site for the period from September 2011 to August 2013, when the 2012–2013 trial was harvested, is plotted in Fig.4D. The plot illustrates the contrast in particular between the periods from April to August, during which 467 mm of rain fell in 2012 and 209 mm in 2013. Drought stress has been shown to increase free asparagine concentration in potato tubers (Muttucumaru, Powers, Elmore, Mottram, & Halford, 2015), although it also causes a decrease in the concentration of reducing sugars, and with the latter being the more important parameter for acrylamide-forming potential in potato, it actually decreases that as well. However, the rainfall at the Woburn site in 2013 was not exceptionally low and there was no evidence that the wheat was drought stressed.

## 4 Conclusions

The study identified eight varieties from the 73 that were included in the study that have shown consistently low free asparagine concentration in the grain in field trials conducted for this study and in trials at different locations in different years in the UK. These were Claire, Cocoon, Cordiale, Croft, Delphi, Horatio, Monterey and Myriad. Cordiale is a Group 2 wheat (breadmaking potential), while Croft, Claire, Cocoon, and Monterey are Group 3 (soft, biscuit type), and Horatio and Myriad are soft Group 4 types (mainly for animal feed and bioethanol, but also used in some food grists). Clearly there is a predominance of soft wheats on this list, but all four groups contained varieties with a wide range of free asparagine concentration and our analysis did not reveal significant differences in free asparagine between the groups overall (p = 0.133, F-test). Choosing varieties for low acrylamide-forming potential simply on the basis of their classification as soft is therefore simplistic and probably ineffective, although there is anecdotal evidence from food industry sources that this is common practice.

It should be noted that the 2012–2013 trial included varieties Evoke, Twister, Goldengun, Bonham, Icon, Dali, Solace, Skyfall, Icebreaker and Lancaster that were new varieties in the process of being introduced to the UK market at the time. Of these, Bonham (G2), Evoke (G2), Goldengun (G4 hard) and Twister (G4 soft) all had relatively low free asparagine concentration (Fig. 2, Fig. 3), but data from other trials were not available to enable the consistency of these varieties to be assessed. The same was true for Cubanita, which had the lowest free asparagine concentration of all the varieties in the 2012–2013 field trial. Nevertheless, the fact that varieties with consistently low free asparagine concentration over multiple field trials are beginning to be identified is consistent with the conclusion of Corol et al. (2016) that some genotypes within the Healthgrain population were more stable with respect to free asparagine concentration than others over multiple years and trial sites.

The annual introduction of multiple new varieties to the market in the UK and the dropping of others from the AHDB Recommended List (https://cereals.ahdb.org.uk/varieties/ahdb-recommended-lists.aspx) is indicative of the competitive market faced by wheat breeders, but may be a problem for end users when it comes to assessing the suitability of different varieties for their products. There is no cheap, rapid test for free asparagine concentration in wheat grain or other crop products, meaning that quality control at the factory gate is not currently feasible. The food industry therefore requires varieties that can be relied upon to produce grain that is consistently low in free asparagine concentration over a range of environments and harvest years, yet by the time this has been established the variety may already have been dropped from the AHDB Recommended List. Cocoon, for example, which is one of the varieties identified in this study as being consistently low in free asparagine concentration, first appeared on the AHDB Recommended List for the 2011–2012 season but only remained on the list for three seasons before being dropped for the 2015–2016 season. The only solution to this problem is for free asparagine concentration to be measured during variety development and in the AHDB’s Recommended List trials, and the data made available to end users when the variety is launched. So far, AHDB has not requested breeders to include data on free asparagine concentration in Recommended List applications and end users do not have access to this information.

Another variety that has shown consistently low free asparagine concentration is Claire. In contrast to Cocoon, this variety has shown great longevity, first appearing on the AHDB Recommended List in 1999 and remaining there to this day (https://cereals.ahdb.org.uk/varieties/ahdb-recommended-lists.aspx). However, its popularity has declined in recent years due to its susceptibility to emerging strains of Yellow rust (*Puccinia striiformis*), which have resulted in AHDB reducing its Yellow rust resistance score from 9 when it was first introduced to 5 (scale 1–9). Claire’s declining popularity has coincided with a striking reduction in the cultivation of Group 3 biscuit wheats as a whole in the UK, falling from approximately 45% of the total area of UK wheat cultivation in 2006 to less than 10% in 2015 (Lockwood, 2015). This trend will alarm biscuit, breakfast cereal and other food manufacturers who require Group 3 types as their preferred raw material, particularly given that five of the varieties that we have identified as being consistently low in free asparagine concentration fall into that group.

Another aspect of Claire’s increasing susceptibility to Yellow rust is that pathogen infection has been linked with an increase in free asparagine concentration in the grain (Curtis et al., 2016). The erosion of a variety’s disease resistance is therefore likely to undermine its consistency with respect to free asparagine concentration, or at least make that consistency more dependent on effective disease control measures.

This study has added to evidence that ensuring that wheat has a plentiful supply of sulphur is a key crop management tool for controlling the potential for acrylamide formation in wheat products. While this is now well-established (Curtis et al., 2014, Curtis et al., 2009, Granvogl et al., 2007, Muttucumaru et al., 2006), the present study identified another important aspect of this in the differential responses of the varieties to sulphur deficiency. Any ranking of varieties according to free asparagine concentration, within or regardless of type, broke down when sulphur supply was deficient. Optimal crop management is therefore at least as important as variety selection for ensuring that the acrylamide-forming potential of wheat is as low as possible, and farmers will have to ensure sulphur sufficiency for their wheat and probably effective disease control as well to get the benefit of investing in a low asparagine variety. Even so, the contrast in free asparagine concentration in the grain from the two trials is a reminder that some factors are outside the control of producers and, therefore, the rest of the supply chain.
